# Additive Potentiation of R334W-CFTR Function by Novel Small Molecules

**DOI:** 10.3390/jpm13010102

**Published:** 2023-01-01

**Authors:** Mafalda Bacalhau, Filipa C. Ferreira, Iris A. L. Silva, Camilla D. Buarque, Margarida D. Amaral, Miquéias Lopes-Pacheco

**Affiliations:** 1 Biosystems & Integrative Sciences Institute, Faculty of Sciences, University of Lisbon, 1749-016 Lisbon, Portugal; 2 Department of Chemistry, Pontifical Catholic University of Rio de Janeiro, Rio de Janeiro 22541-041, Brazil

**Keywords:** CFTR modulator, co-potentiation, cystic fibrosis, drug discovery, intestinal organoids, potentiator, rare mutation

## Abstract

The R334W (c.1000C>T, p.Arg334Trp) is a rare cystic fibrosis (CF)-causing mutation for which no causal therapy is currently approved. This mutation leads to a significant reduction of CF transmembrane conductance regulator (CFTR) channel conductance that still allows for residual function. Potentiators are small molecules that interact with CFTR protein at the plasma membrane to enhance CFTR-dependent chloride secretion, representing thus pharmacotherapies targeting the root cause of the disease. Here, we generated a new CF bronchial epithelial (CFBE) cell line to screen a collection of compounds and identify novel potentiators for R334W-CFTR. The active compounds were then validated by electrophysiological assays and their additive effects in combination with VX-770, genistein, or VX-445 were exploited in this cell line and further confirmed in intestinal organoids. Four compounds (LSO-24, LSO-25, LSO-38, and LSO-77) were active in the functional primary screen and their ability to enhance R334W-CFTR-dependent chloride secretion was confirmed using electrophysiological measurements. *In silico* ADME analyses demonstrated that these compounds follow Lipinski’s rule of five and are thus suggested to be orally bioavailable. Dose–response relationships revealed nevertheless suboptimal efficacy and weak potency exerted by these compounds. VX-770 and genistein also displayed a small potentiation of R334W-CFTR function, while VX-445 demonstrated no potentiator activity for this mutation. In the R334W-expressing cell line, CFTR function was further enhanced by the combination of LSO-24, LSO-25, LSO-38, or LSO-77 with VX-770, but not with genistein. The efficacy of potentiator VX-770 combined with active LSO compounds was further confirmed in intestinal organoids (R334W/R334W genotype). Taken together, these molecules were demonstrated to potentiate R334W-CFTR function by a different mechanism than that of VX-770. They may provide a feasible starting point for the design of analogs with improved CFTR-potentiator activity.

## 1. Introduction

Cystic fibrosis (CF) is a life-shortening autosomal recessive inherited disease that leads to a multiorgan pathology [1,2]. It is caused by mutations in the gene encoding the CF transmembrane conductance regulator (CFTR) protein, an ATP-gated chloride channel activated by protein kinase A (PKA)-dependent phosphorylation [2,3]. CFTR is expressed at the apical membrane of secretory epithelia and plays a fundamental role in fluid and electrolyte movements, thus controlling the composition and amount of epithelial secretions [3,4]. In the airways, loss of CFTR function causes depletion of periciliary liquid and mucus accumulation that along with chronic inflammation and recurrent infections progressively impair lung function [3,4].

Over the last decade, CF therapies have been transformed by the approval of orally bioavailable drugs targeting the root cause of disease—so-called CFTR modulators [2]. The potentiator VX-770 (also termed ivacaftor) was the first CFTR-modulator drug approved for clinical use initially for people with CF (PwCF) carrying the G551D mutation [5]. Subsequent studies led to label extensions of VX-770 monotherapy for several other gating and residual function mutations [6,7,8,9,10]. VX-770 acts by increasing the function of mutant CFTR channels present at the plasma membrane [11]. Once CFTR is phosphorylated by PKA, VX-770 enhances the duration and frequency of channel opening in an ATP-independent channel gating, thus allowing for CFTR-dependent chloride secretion [12]. Clinically, VX-770 monotherapy has been demonstrated to promote sustained and long-term clinical benefits, including a slower decline of lung function and improved nutritional status [13,14]. When combined with CFTR correctors, which rescue CFTR folding and trafficking to the plasma membrane, VX-770 also induced therapeutic benefits for PwCF carrying the most prevalent F508del mutation [15,16,17].

Despite significant therapeutic progress, many PwCF carrying rare mutations remain without modulator therapy approved [2,4]. The R334W mutation (c.1000C>T, p.Arg334Trp) is found in ~0.3% of CF alleles worldwide (https://cftr2.org, accessed on 10 October 2022), but has a relatively higher prevalence in Portuguese and Brazilian CF populations (2.8% and 2.3% of CF alleles, respectively) [18,19]. This mutation has minimal impact on CFTR folding and trafficking, but significantly affects chloride distribution at the mouth of the channel pore, thus resulting in either very low channel conductance or reduced channel open probability that still enables a residual CFTR-dependent chloride secretion [20,21]. In cell models expressing R334W-CFTR, the results have been conflicting with respect to the effects of VX-770 [7,22,23]. While no significant response to VX-770 was detected in experiments using Fischer rat thyroid (FRT) cells expressing this mutant [7,22], a small but significant increase in CFTR function was observed in R334W-expressing CF bronchial epithelial (CFBE) cells [23]. Furthermore, VX-770 does not fully restore CFTR gating defects in G551D- and F508del-expressing cells [11,24], which indicates that there is still scope for further enhancement. Recent reports have found that a combination of potentiators with complementary mechanisms (i.e., co-potentiators) can further enhance CFTR-dependent chloride secretion [22,23,25,26], and several small molecules with a chemical structure distinct from that of VX-770 have been demonstrated to rescue the function of mutant CFTR channels at different potencies and degrees of efficacy [22,27,28,29].

Triazoles are well-known synthetic heterocycle compounds widely used in both industry and academic research [30,31]. This scaffold can be obtained by various synthetic routes, although the copper-catalyzed azide–alkyne click chemistry reaction is the most commonly applied due to its simplicity, robustness, and versatility [30,31]. The 1,4-disubstituted 1,2,3-triazole pharmacophoric group has been used in the design of drugs with antimicrobial [32], anti-inflammatory [33], anti-viral [34], and anti-tumor properties [35,36,37], proving to be a successful strategy in medicinal chemistry and capable of mimicking the characteristics of different functional groups. In this study, we have generated a new CFBE cell line co-expressing the halide sensitive-yellow fluorescence protein (HS-YFP) and R334W-CFTR to screen a collection of triazole compounds and derivatives in order to identify novel potentiators. The active compounds were validated by electrophysiological measurements and their potential utility as co-potentiators with VX-770, genistein, or VX-445 was exploited in this cell line and further confirmed in intestinal organoids.

## 2. Materials and Methods

### 2.1. Chemicals and Compounds

The triazole compounds and derivatives were synthesized in the “Laboratório de Síntese Orgânica” (LSO) and physiochemically characterized as described previously [37,38]. The remaining compounds were purchased from commercial sources at the analytical grade: VX-770 (#HY-13017), genistein (#HY-14596), forskolin (Fsk, #HY-15371), and CFTR channel inhibitor (CFTRinh-172, #16671) are from MedChemExpress (Monmouth Junction, NJ, USA); while VX-445 (#S8851) is from Selleckchem (Houston, TX, USA). All compounds were diluted in dimethyl sulfoxide (DMSO).

### 2.2. Generation of a New Cell Line

A new CFBE cell line stably co-expressing the HS-YFP (F46L/H148Q/I152L) and R334W-CFTR was generated. Briefly, the HS-YFP cloned into the pcDNA3.1 expression vector was re-cloned into the lentiviral expression vector pLVX-Puro and then transfected into the HEK-293T cells to produce the lentiviral particles [39]. After 48 h, these particles were harvested and transduced into CFBE cells expressing R334W-CFTR [40]. The efficiency of transduction was assessed by fluorescence microscopy (Zeiss Axiovert 200, Jena, Germany) and then cells were sorted in a FACSAria III cell sorter (BD Biosciences, Franklin Lakes, NJ, USA) to select a homogeneous population with the highest expression of the HS-YFP.

### 2.3. Cell Culture

CFBE cell lines expressing CFTR variants (WT and R334W) with and without co-expression of the HS-YFP were cultured in minimum essential medium (MEM, #10-010-CV, Corning, VA, USA) supplemented with 10% fetal bovine serum (FBS, #LTI 10270-106, Gibco, Carlsbad, CA, USA) and 2 µg.mL^-1^ puromycin (#P8833, Sigma-Aldrich, St. Louis, MO, USA) [40,41]. All cells were maintained at 37 °C and 5% CO_2_ in a humidified incubator, except for the low-temperature experiments, in which cells were cultured at 27 °C for 24 h.

### 2.4. HS-YFP Assay on a Plate Reader

A microplate reader (Tecan Infinite 200 Pro) equipped with high-quality excitation (485 ± 20 ηm) and emission (535 ± 25 ηm) was used for compound screening [40]. Briefly, CFBE cells co-expressing the HS-YFP and R334W-CFTR were plated in 96 well black-walled, clear-bottom microplates (#655090, Greiner Bio-One, Kremsmünster, Austria) at a density of 50,000 cell/well. On the following day, cells were washed twice with phosphate-buffered saline (PBS) and incubated for 30 min with 60 µL PBS with Fsk (5 µM) and test compounds. All plates in the potentiator screen contained wells with Fsk (5 µM) plus positive (1 µM VX-770 or 50 µM Gen) or negative (DMSO) controls. For the co-potentiator analysis, CFBE cells co-expressing CFTR variants were incubated with Fsk (5 µM) plus VX-770 (1 µM), Gen (50 µM), or VX-445 (3 µM) and active LSO compounds for 30 min. The assay consisted of 14-s fluorescence readings: 2-s of the initial fluorescence intensity and 12-s after injection of 100 µL of an iodide-containing solution (PBS with NaCl substituted by NaI, final iodide concentration: 100 mM). The initial iodide influx was computed from fluorescence intensity by single exponential regression [42]. All conditions were performed in triplicate in each microplate.

### 2.5. In Silico Absorption, Distribution, Metabolism, and Excretion (ADME) Analyses

ADME analyses were carried out using the free online software SwissADME (http://www.swissadme.ch, accessed on 10 October 2022) as previously described [43]. The following physicochemical variables were assessed according to Lipinski’s rule of five [44]: molecular weight (MW); number of H-bond donors (nHBD); number of H-bond acceptors (nHBA); number of rotatable bonds (nRB); the calculated logarithm of the octanol-water partition coefficient (LogP); in addition to the topological polar surface area (TPSA). Active compounds were also subjected to filters for the identification of pan-assay interference compounds (PAINS), i.e., “promiscuous” substructures that demonstrate potential response in bioassays independent of the target [45].

### 2.6. WB Analyses

For CFTR-protein detection, CFBE cells were lysed using a lysis buffer (31.25 mM Tris-HCl pH 6.8, 1.5% SDS [*w*/*v*], 5% glycerol and 0.5 mM DTT) supplemented with a complete protease inhibitor cocktail (#11697498001, Roche, Basel, Switzerland) and Laemmli sample buffer (#1610747, Bio-Rad, Hercules, CA, USA). Whole-cell lysates were subjected to SDS-PAGE and transferred to a polyvinylidene fluoride membrane (#88518, Millipore, Burlington, MA, USA). CFTR was detected using the monoclonal anti-human CFTR antibody 596 (1:3,000, CF Foundation Therapeutics) and the blotting-grade horseradish peroxidase secondary antibody (1:5,000, Bio-Rad, Hercules, CA, USA). Calnexin (Clnx) was detected using the monoclonal anti-calnexin antibody (1:3,000, #610523, BD Biosciences, Franklin Lakes, NJ, USA) as a loading control. Images were acquired using the ChemiDoc XRS+ imaging system and analyzed with the Image lab version 6.0 (Bio-Rad, Hercules, CA, USA).

### 2.7. Micro-Ussing Chamber Measurements

Short-circuit current was measured on polarized CFBE cells expressing R334W or WT-CFTR cultured on porous membrane filters (#734-1646, Corning, New York, NJ, USA) as described [46,47]. Briefly, the transepithelial electrical resistance (TEER) of cells was measured with the Chopstick Electrode (STX2, WPI, Sarasota, FL, USA), and when it was ≥1,000 Ω.cm^2^, recordings were carried out in modified micro-Ussing chambers. The solution bathing the basolateral membrane contained (in mM) 145 NaCl, 1.6 K_2_HPO_4_, 5.0 D-glucose, 1.0 MgCl_2,_ and 1.3 Ca-gluconate. The solution bathing the apical membrane contained (in mM) 32 NaCl, 0.4 KH_2_PO_4_, 1.6 K_2_HPO_4_, 5.0 D-glucose, 1.0 MgCl_2_, 5.7 Ca-gluconate, and 112 Na-glucose. After the equilibration period, baseline values were recorded and compounds were added sequentially: Fsk (0.128 µM), test compound, and CFTRinh-172 (30 µM). Transepithelial resistance (R_te_) and the voltage (V_te_) were recorded, and equivalent cAMP-stimulated CFTR short-circuit currents (Isc_eq_) were calculated by Ohm’s law from R_te_ and V_te_ as follows: Isc_eq_ = V_te_/R_te_.

### 2.8. Fsk-Induced Swelling (FIS) Assay of Intestinal Organoids

Intestinal organoids were seeded in flat-bottom 96 well microplates in 4 µL of matrigel (#356231, Corning, New York, NJ, USA) and 50 µL of culture medium [48]. After 24 h, organoids were incubated for 20 min with 3 µM of calcein green (#C3100MP, Invitrogen, Waltham, MA, USA) and before imaging, organoids were incubated with Fsk at different concentrations (0.02, 0.128, 0.8, and 5 µM) with and without test compounds as described [48]. Images were acquired every 10 min for 60 min in confocal live-cell microscopy (Leica SP8, Leica Microsystems, Wetzlar, Germany) at 37 °C with 5% CO_2_. FIS was quantified using a CellProfiler-based algorithm and expressed as the absolute area under the curve (AUC) calculated from the normalized surface-area increase (baseline = 100%, *t* = 60 min). 

### 2.9. Statistical Analyses

All conditions were carried out in at least three independent experiments. Data are represented as mean + standard deviation (SD), and dots depict value for each replicate. GraphPad Prism software version 8.3 (GraphPad Inc., San Diego, CA, USA) was used for all statistical analyses. Statistical comparisons between the conditions tested were assessed using one-way ANOVA followed by Dunnett’s or Tukey’s posthoc tests, and values of *p* < 0.05 were considered significant.

## 3. Results

### 3.1. Characterization of a New Cell Line Co-Expressing the HS-YFP and R334W-CFTR 

**Figure 1:**
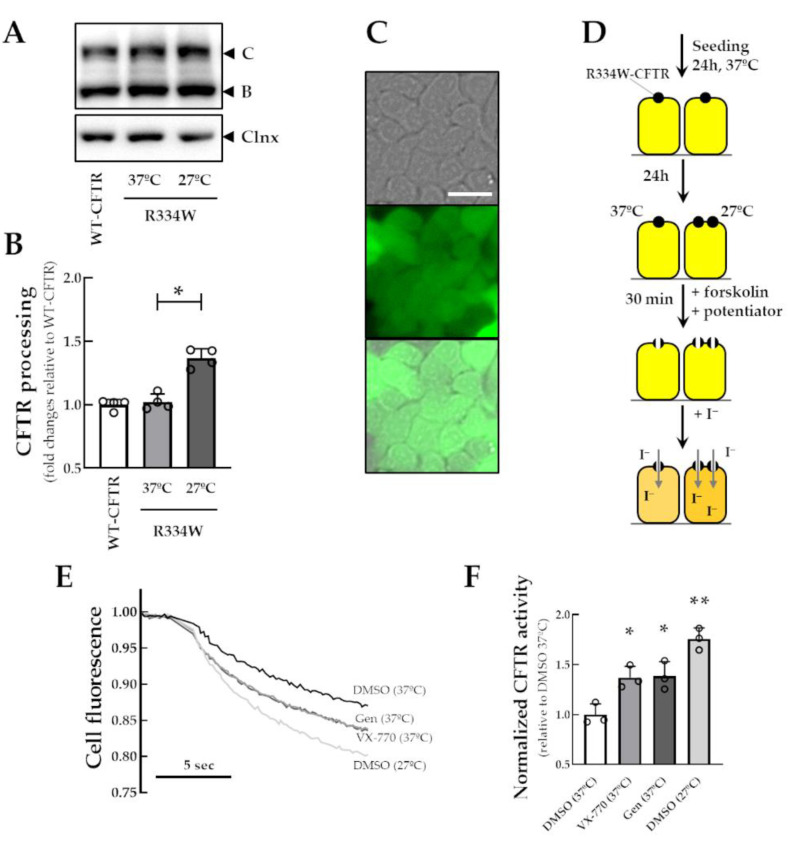
**Characterization of a new cell line co-expressing the HS-YFP and R334W-CFTR.** (**A**) Representative WB images of CFBE cells expressing WT- and R334W-CFTR incubated for 24 h at 37 °C or 27 °C. (**B**) CFTR processing [band C/bands B+C] was quantified and normalized to calnexin (Clnx) levels (loading control). Data are represented as means + SD: * *p* < 0.05 vs. R334W 37 °C. (**C**) R334W-CFTR-expressing CFBE cells demonstrating cytosolic fluorescence of the HS-YFP (F46L/H148Q/I152L). Scale bar: 50 µm. (**D**) Schematic flow of the HS-YFP assay. CFBE cells co-expressing R334W-CFTR and the HS-YFP were incubated for 24 h at 37 °C or 27 ° C and then acutely stimulated (30 min) with Fsk (5 µM) plus DMSO (negative control), VX-770 (1 µM) or Gen (50 µM). (**E**) Representative cell fluorescence recording on a plate reader. (**F**) CFTR function was assessed based on the cell fluorescence quenching rate and normalized to the negative control. Data are represented as means + SD: * *p* < 0.05 and ** *p* < 0.01 vs. DMSO (37 °C).

The R334 residue is located on transmembrane segment 6 at the outer mouth of the CFTR channel pore (Figure S1). Compared to WT-CFTR, the R334W mutation similarly exhibited the presence of both core-glycosylated immature (~140 kDa, band C) and fully-glycosylated mature (~180 kDa, band C) forms of CFTR. Furthermore, R334W-CFTR processing was increased by the incubation of cells at low temperature (1.35-fold increase) (Figure 1A,B).

Bright-field, fluorescence, and merged images were acquired to confirm the transduction of the YFP sensor in CFBE cells stably expressing R334W-CFTR (Figure 1C). These cells were then used for the functional assay to measure the HS-YFP quenching following the addition of iodide-containing solution to the apical surface of cells (Figure 1D). A decay in cell fluorescence was observed upon Fsk stimulation, which was further increased by Fsk stimulation together with VX-770 or genistein (1.37- and 1.38-fold increase, respectively) (Figure 1E,F). Cells incubated at 27 °C for 24 h also demonstrated an increase in cell fluorescence quenching upon Fsk stimulation compared to those cultured at 37 °C (1.76-fold increase).

### 3.2. Identification of Novel Potentiators for the Rescue of R334W-CFTR Function

**Figure 2:**
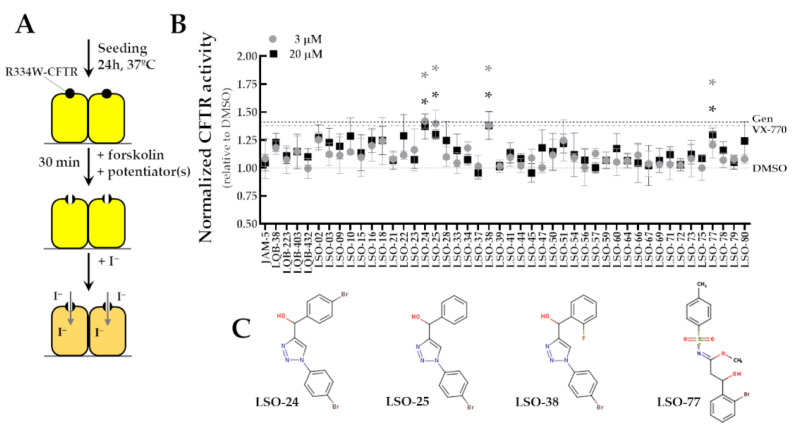
**Search of novel R334W-CFTR potentiators by a high-throughput screening assay.** (**A**) Schematic flow of the HS-YFP assay. CFBE cells co-expressing R334W-CFTR and the HS-YFP were acutely stimulated (30 min) with Fsk (5 µM) plus test compound. DMSO, VX-770 (1 µM), and Gen (50 µM) were used as negative and positive controls, respectively. (**B**) CFTR function was assessed based on the cell-fluorescence quenching rate and normalized to the negative control. Data are represented as means + SD of 4 independent experiments: * *p* < 0.05 vs. DMSO. (**C**) Chemical structure of active compounds: LSO-24, LSO-25, LSO-38, and LSO-77.

To assess the potential utility of the investigational compounds as novel potentiators for R334W-CFTR, cells were acutely incubated (30 min) with a test compound together with Fsk before assay (Figure 2A). Among 46 compounds, four demonstrated a small increase, albeit significant, in the HS-YFP quenching rate compared to DMSO (1.21- to 1.42-fold increase) (Figure 2B). Furthermore, the effect of LSO-24, LSO-25, LSO-38, and LSO-77 was closely similar to that of VX-770 or genistein. Figure 2C depicts the chemical structure of the active compounds in the screening. The efficacy and potency of these compounds were further assessed by the incubation of cells with various concentrations in the range of 0.03 to 30 µM and the HS-YFP quenching rate was measured (Figure S2). Table 1 depicts EC_50_ and E_max_ for these compounds.

The theoretical ADME properties of the active LSO compounds were determined by *in silico* analysis according to Lipinski’s rule of five, which states that a small molecule should have MW ≤ 500 Da, nHBD ≤ 5, nHBA ≤ 5, nRB ≤ 10, and LogP ≤ 5 to be orally active. TPSA should also be ≤ 140 Å^2^, since it influences absorption and membrane permeability. Table 2 depicts the values for compounds LSO-24, LSO-25, LSO-38, and LSO-77, in addition to those for the reference compound VX-770. All four LSO compounds obey Lipinski’s rule of five and have a TPSA < 140 Å^2^, suggesting they can be well absorbed in the gastrointestinal tract. Moreover, during the *in silico* analysis, these compounds were not recognized as PAINS.

**Figure 3:**
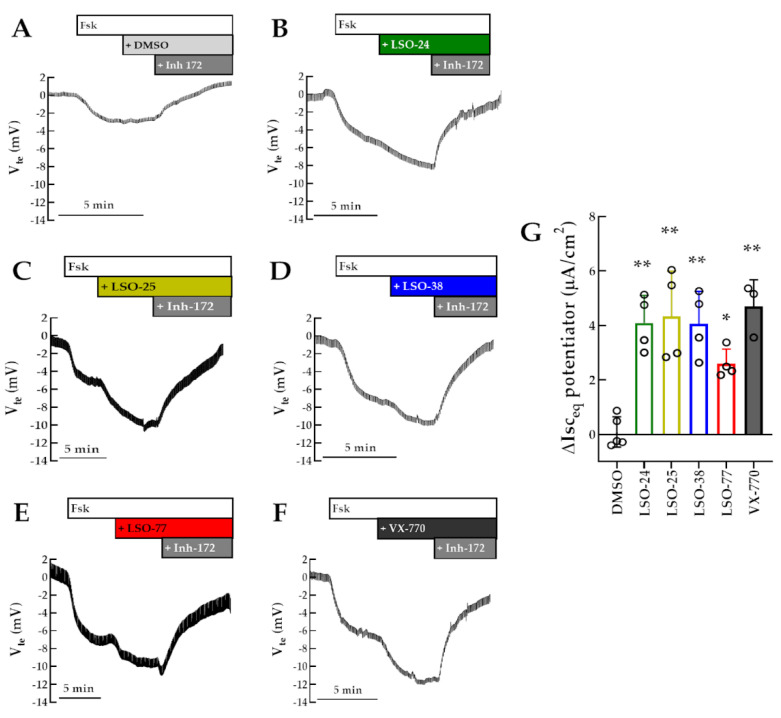
**Assessment of compounds on R334W-CFTR function by micro-Ussing chamber measurements.** (**A**–**F**) Representative Ussing chamber (open-circuit) recording representing transepithelial voltage measurements (V_te_) of polarized monolayers of CFBE cells expressing R334W-CFTR. Cells were acutely subsequently stimulated with Fsk (0.128 µM), test compounds (DMSO, 5 µM LSO-24, 5 µM LSO-25, 5 µM LSO-38, 10 µM LSO-77, or 3 µM VX-770) and CFTRInh-172 (30 µM). (**G**) Data are represented as mean (+ SD) increase in I_eq_ promoted by test potentiator: * *p* < 0.05 and ** *p* < 0.01 vs. DMSO.

To validate the results obtained by the HS-YFP assay on a plate reader, short-circuit current was measured in polarized monolayers of CFBE cells expressing R334W-CFTR to quantitatively assess CFTR-dependent chloride current (Figure 3). Activation of cAMP-dependent CFTR-mediated chloride secretion by Fsk promoted a small increase in equivalent short-circuit current (Isc_eq_), which was further increased by the subsequent addition of the potentiator LSO-24, LSO-25, LSO-38, LSO-77, or VX-770 (4.1, 4.3, 4.1, 2.6 and 4.7 µA/cm^2^, respectively). These responses were thus inhibited by the addition of CFTRinh-172, indicating they are CFTR-specific.

### 3.3. Assessment of Potentiator Activity for WT-CFTR

**Figure 4:**
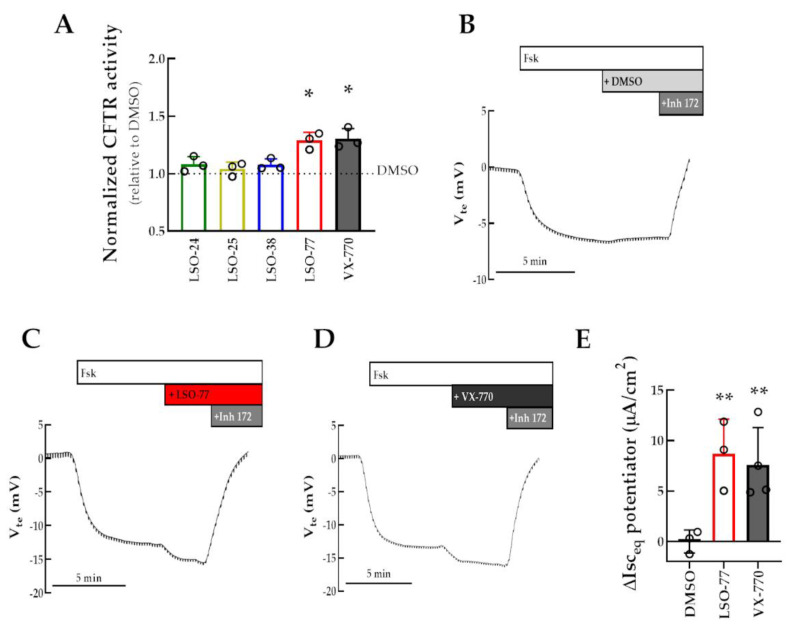
**Assessment of compounds on WT-CFTR function.** (**A**) CFBE cells co-expressing WT-CFTR and the HS-YFP were acutely stimulated (30 min) with Fsk (5 µM) plus test compound: LSO-24; 5 µM LSO-25; 5 µM LSO-38; or 10 µM LSO-77. DMSO and VX-770 (1 µM) were used as negative and positive controls, respectively. CFTR function was assessed based on the cell-fluorescence quenching rate and normalized to the negative control. Data are represented as means + SD: * *p* < 0.05 vs. DMSO. (**B**–**D**) Representative Ussing-chamber (open-circuit) recording representing transepithelial voltage measurements (V_te_) of polarized monolayers of CFBE cells expressing WT-CFTR. Cells were acutely subsequently stimulated with Fsk (0.128 µM), test compounds (DMSO, 10 µM LSO-77, or 3 µM VX-770), and CFTRInh-172 (30 µM). (**E**) Data are represented as mean (+SD) increase in I_eq_ promoted by test potentiator: * *p* < 0.05, ** *p* < 0.01 vs. DMSO.

Next, we assessed the ability of active LSO compounds in potentiating WT-CFTR function. In the HS-YFP assay on a plate reader, a further increase in cell-fluorescence quenching rate was observed in WT-CFTR-expressing cells incubated with LSO-77 or VX-770 (1.30- and 1.31-fold increase, respectively) compared to those incubated with DMSO (Figure 4A). These results were also confirmed in polarized monolayers of CFBE cells expressing WT-CFTR in the Ussing chamber. A negative deflection was observed upon Fsk stimulation that was further increased following the acute application of LSO-77 or VX-770 (Figure 4B–E). However, LSO-24, LSO-25, and LSO-38 were unable to potentiate WT-CFTR function either in the HS-YFP assay on a plate reader (Figure 4A) or in Ussing-chamber measurements (Figure S3).

### 3.4. Assessment of Co-Potentiator Activity for R334W-CFTR in a Cell Line and in Intestinal Organoids

**Figure 5:**
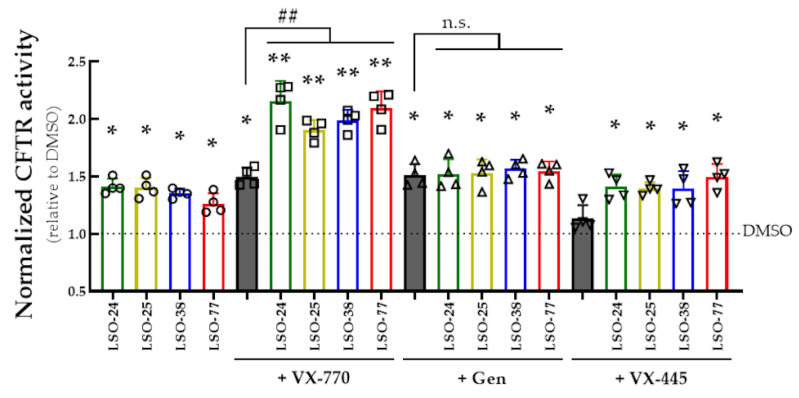
**Assessment of additivity of potentiators activity on R334W-CFTR function in a cell line.** CFBE cells co-expressing R334W-CFTR and the HS-YFP were acutely stimulated (30 min) with Fsk (5 µM) plus test compounds (individually or in combination): LSO-24 (5 µM); LSO-25 (5 µM); LSO-38 (5 µM); LSO-77 (10 µM); VX-770 (1 µM); Gen (50 µM); and VX-445 (3 µM). CFTR function was assessed based on the cell-fluorescence quenching rate and normalized to the negative control (DMSO). Data are represented as means + SD: * *p* < 0.05 and ** *p* < 0.01 vs. DMSO. Other symbols indicate the statistical significance of groups of data vs. VX-770 alone: ^##^ *p* < 0.01; n.s.—not significant.

In order to investigate the additivity of potentiator activity for the LSO compounds, cells were acutely incubated (30 min) with LSO-24, LSO-25, LSO-38, and LSO-77 together with VX-770, genistein or VX-445 and the HS-YFP assay on a plate reader was carried out (similarly to the scheme presented in Figure 2A). Upon Fsk stimulation, an increase in fluorescence quenching rate was observed in cells acutely treated with VX-770 in combination with LSO-24, LSO-25, LSO-38, or LSO-77 compared to DMSO (1.91- to 2.15-fold increase) or any compound individually (Figure 5). However, the combination of LSO compounds with genistein demonstrated an equivalent cell-fluorescence quenching rate to that of genistein alone. VX-445 was unable to increase the cell-fluorescence quenching rate compared to DMSO, and its combination with LSO-24, LSO-25, LSO-38, or LSO-77 displayed effects similar to those of each compound individually (Figure 5).

**Figure 6:**
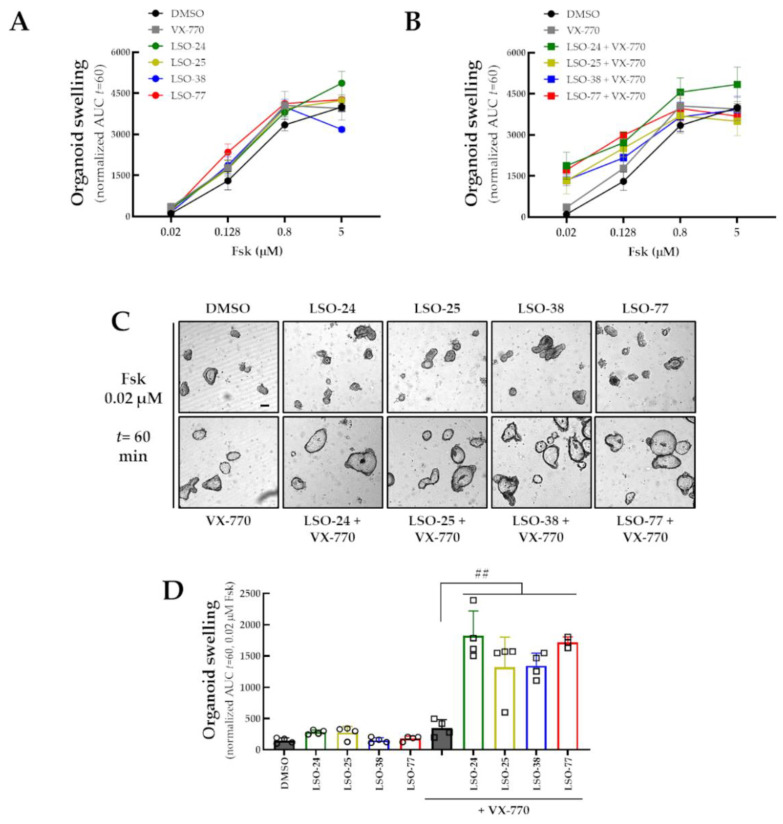
**Assessment of additivity of potentiators activity on R334W-CFTR function in intestinal organoids.** (**A**,**B**) Quantification of FIS in intestinal organoids from an individual with CF (R334W/R334W genotype) for all test compounds (**A**) individually and (**B**) in combination with VX-770 at Fsk concentrations of 0.02, 0.128, 0.8, and 5 µM, expressed as the AUC of organoid surface area increase (baseline = 100%, *t* = 60 min). (**C**) Representative images of intestinal organoids (Scale bar: 50 µm) and (**D**) quantification at 0.02 µM Fsk for test compounds individually or in combination. Data are represented as means + SD: ^##^ *p* < 0.01 vs. VX-770 alone.

The ability of LSO-24, LSO-25, LSO-38, and LSO-77 with and without VX-770 in potentiating R334W-CFTR function was further assessed using the FIS assay of intestinal organoids from an individual with CF (R334W/R334W genotype) (Figure 6A,B). The incremental Fsk concentration in organoids acutely incubated with DMSO led to increasing FIS values, compatible with a residual function in R334W-CFTR. Upon stimulation with 0.128 µM Fsk plus LSO-24, LSO-25, LSO-38, LSO-77, or VX-770, organoid FIS values demonstrated a small but significant increase compared to that of DMSO (1.32- to 1.81-fold increase) (Figure 6A,B). On the other hand, organoid FIS values were similar to those of DMSO upon stimulation with 0.02 µM Fsk with any single potentiator (Figure 6C,D). However, significantly higher FIS values were observed for organoids incubated with LSO-24, LSO-25, LSO-38, or LSO-77 in combination with VX-770, compared to DMSO (3.78- to 5.36-fold increase vs. VX-770 alone), upon stimulation with 0.02 µM Fsk (Figure 6C,D).

## 4. Discussion

This study aimed to screen a collection of compounds to identify novel potentiators for R334W-CFTR, a residual function mutation that is reported to have no to suboptimal susceptibility to VX-770 monotherapy [7,22,23]. Because the four active compounds identified in the functional primary screening only demonstrated a small potentiation of R334W-CFTR function, their additivity with VX-770, genistein, or VX-445 was assessed in a new cell line and then further validated in intestinal organoids. Moreover, LSO-24, LSO-25, LSO-38, and LSO-77 were found to obey Lipinski’s rule of five for drug-like molecules [44] and are thus suggested to have good bioavailability.

Similar to WT-CFTR, the R334W mutation enables CFTR to be fully glycosylated and traffic to the plasma membrane. However, in contrast to WT-CFTR, which is a functional protein, R334W-CFTR presents a significantly reduced channel conductance [20,21]. When R334W-expressing cells were shifted from 37 °C to 27 °C, there was an increase in CFTR processing, indicating that this mutant is temperature sensitive, similar to results previously reported for WT- and F508del-CFTR [49,50,51]. Furthermore, the increased cell-fluorescence quenching rate suggests that low-temperature incubation resulted in higher R334W-CFTR-dependent anion transport, most likely by increasing the number of CFTR channels at the plasma membrane rather than enhancing channel gating.

Numerous screening campaigns have been carried out to identify CFTR potentiators in libraries of drug-like small molecules, which have resulted in the discovery of several compounds with distinct chemical structures that are able to potentiate CFTR function [11,25,27,29,52]. Nevertheless, few potentiators achieved clinical investigation and up to recently, only the potentiator VX-770 was clinically approved for PwCF carrying specific mutations [5,6,7,8,9,10]. ABBV-974 (formerly GLPG-1837) has the same binding site as VX-770 in the transmembrane domains of CFTR [53,54] and acts by facilitating the channel gating in a phosphorylation-dependent and ATP-independent fashion [12,55]. GLPG-2451 has improved potency [52] but is expected to share the same mechanism as VX-770 and ABBV-974 [27]. A previous study attempted to substitute the amide bioisostere in the VX-770 structure for a 1,2,3-triazole scaffold and demonstrated reduced efficacy and potency in potentiating WT-, F508del- and G551D-CFTR function by this compound series [56]. The active triazole compounds identified in our functional screening (LSO-24, LSO-25, and LSO-38) also demonstrated a suboptimal efficacy and weak potency, but they appear to potentiate R334W-CFTR function by a different mechanism than that of VX-770. Intriguingly, LSO-77, but not LSO-24, LSO-25, or LSO-38, was able to potentiate WT-CFTR function. This compound has an *N*-tosylpropanimidate instead of a triazole in the chemical structure, which might confer some different pharmacological properties compared to the other three active compounds. Genistein and its analog apigenin also potentiate CFTR channel gating by a distinct mechanism to that of VX-770 [25,29]. They are presumed to bind at the nucleotide-binding domain 1 and 2 interface and promote dimerization, thus accelerating channel opening and slowing down its closure [29]. ASP-11 is another compound that is suggested to potentiate CFTR function by this mechanism [25]. Furthermore, VX-445 (also termed elexacaftor) was initially identified as a CFTR corrector and approved for clinical use in the triple-combo therapy VX-445/VX-661/VX-770 [17], but is now recognized as a compound with dual corrector and potentiator activity [26,57]. Although the mechanism by which VX-445 promotes CFTR potentiation has not been elucidated, it is suggested to be different from that exerted by VX-770 and apigenin [26,58].

Despite the therapeutic success of VX-770 monotherapy [5], it achieves only a partial restoration of the CFTR-gating activity for several mutations, including G551D and F508del [11,24,26], and PwCF still experience a progressive decline of their lung function and pulmonary exacerbations albeit at reduced frequency [14,59]. As different potentiators may promote distinct conformational alterations in the CFTR protein to increase channel gating/conductance [53,54], co-potentiators have emerged as a strategy to enhance CFTR-dependent chloride secretion for mutations that do not respond well to a single potentiator [22,23,25,26]. Combinatorial profiling has shed some light on the mechanism of co-potentiation by clustering compounds into three mechanistic classes [22,26]. Nevertheless, CFTR mutations are not equally responsive to combined potentiators and some mutations were demonstrated to be sensitive (e.g., G551D, G1244E, and S1251N) while others were unresponsive (e.g., R347P, V520F, and L1077P) [22,23,26]. N1303K-CFTR function was also demonstrated to synergistically respond to a triple potentiator combo (VX-770, apigenin, and VX-445) [58]. Our investigational compounds (LSO-24, LSO-25, LSO-38, and LSO-77) demonstrated an additive potentiation of R334W-CFTR function in combination with VX-770, but not with genistein. These results suggest that they may share a common mechanism with genistein. ASP-11 is also suggested to share a common mechanism with genistein, but it was not additive to potentiate R334W-CFTR function in combination with VX-770 [22]. This probably occurred because this mutation is associated with residual CFTR function and the usage of a high concentration of Fsk (20 µM) already saturated CFTR activity [22], thus hindering further potentiation of CFTR-channel activity. Indeed, such results were also observed for our investigational compounds in the FIS assay of intestinal organoids (R334W/R334W genotype). At the lowest concentration of Fsk (0.02 µM), there was a clear difference between the negative control and single potentiators with the co-potentiator treatments. However, upon the incremental concentration of Fsk, such differences could no longer be easily detected due to high residual function. Similar behavior was also reported for D614G, another mutation with residual CFTR function [60], reinforcing thus the need for assessing the concentration of Fsk (or other CFTR activators) when investigating a residual function mutation or when testing a ‘highly effective’ modulator combination.

Although the R334W mutation causes neither folding nor trafficking impairment, some studies have demonstrated that R334W-CFTR-dependent chloride secretion can be enhanced by increasing the number of R334W-CFTR channels at the plasma membrane with correctors, such as VX-809 and VX-661 [39,40,46,61]. In a recent report, intestinal organoids from an individual with CF (1677delTA/R334W) were incubated with VX-770, VX-809/VX-770, VX-661/VX-770 and VX-445/VX-661/VX-770 [62]. Upon Fsk stimulation, an increase in FIS values was observed with the different modulator combinations but to a comparable extent to that of VX-770 alone [62]. Another report assessed the efficacy of VX-445/VX-661/VX-770 in F508del/R334W intestinal organoid-derived epithelial monolayers and demonstrated rescue of CFTR-mediated anion transport [63]. However, it has been suggested that the triple-combo therapy only modestly improved R334W-CFTR function and most of the gain in function was attributed to F508del-CFTR rescue [63]. While the corrector activity of VX-445 on R334W-CFTR needs to be further elucidated, our data indicate that this compound does not potentiate R334W-CFTR function when used alone or in combination with LSO molecules. 

## 5. Conclusions

In summary, the present study identified four small molecules that are able to potentiate R334W-CFTR function, a mutation for which no modulator therapy is currently approved. Despite the suboptimal efficacy of LSO-24, LSO-25, LSO-38, and LSO-77, they have different scaffolds from that of previous potentiators and may thus represent a valuable starting point to design analog molecules with improved CFTR potentiator activity. These compounds also demonstrate an additive potentiation of CFTR function in combination with VX-770 in both R334W-heterologously expressing cells and intestinal organoids from a R334W homozygous CF subjects. The additive potentiation effects support the idea that they act by a mechanism distinct from that of VX-770, and their potential utility to enhance CFTR function for other mutations should be exploited in upcoming studies.

## Figures and Tables

**Table 1:** EC_50_ and E_max_ of Active LSO Compounds.

Compound	EC_50_ (µM)	E_max_ (µM)
LSO-24	1.24	3.6
LSO-25	1.41	4.3
LSO-38	1.81	3.7
LSO-77	2.96	6.9

**Table 2:** In Silico Absorption, Distribution, Metabolism and Excretion (ADME) Analysis.

Comp	MW	nHBD	nHBA	nRB	LogP	TPSA (Å)	GI Absorption	PAINS Alert
LSO-24	409.08	1	3	3	3.54	50.94	High	0
LSO-25	330.18	1	3	3	2.77	50.94	High	0
LSO-38	348.17	1	4	3	3.11	50.94	High	0
LSO-77	412.30	1	5	6	3.60	84.34	High	0
VX-770	392.49	3	3	5	4.47	82.19	High	0

Comp—compound; MW—molecular weight; nHBD—number of H-bond donors; nHBA—number of H-bond acceptors; nRB—number of rotatable bonds; LogP—values correspond to Consensus LogP_w/o_; TPSA—topological polar surface area; GI—gastrointestinal; PAINS—pan-assay interference structures.

## Data Availability

The data presented here are available on request from the corresponding author. The data are not publicly available due to privacy and ethical issues.

## Supplementary Materials

The following supporting information can be downloaded at: https://www.mdpi.com/article/10.3390/jpm13010102/s1, Figure S1—Ribbon diagram of the dephosphorylated; ATP-free human CFTR structure; Figure S2—Dose-response relationships for the compounds; Figure S3—Assessment of LSO-24, LSO-25 and LSO-38 on WT-CFTR function.
