# Supplementary material for: Additive Potentiation of R334W-CFTR Function by Novel Small Molecules
Source: J Pers Med. 2023 Jan 1;13(1):102. doi: 10.3390/jpm13010102 (PMC9862739; doi:10.3390/jpm13010102)
Supplement: Supplementary file 1 [file jpm-13-00102-s001.zip › jpm-2041440-supplementary.pdf]

Supplementary Material

Additive Potentiation of R334W-CFTR Function by Novel Small Molecules

Mafalda Bacalhau [1], Filipa C. Ferreira [1], Iris A. L. Silva [1], Camilla D. Buarque [2], Margarida D. Amaral [1] and Miquéias Lopes-Pacheco [1,*]

[1] Biosystems & Integrative Sciences Institute, Faculty of Sciences, University of Lisbon, 1749-016 Lisbon, Portugal
[2] Department of Chemistry, Pontifical Catholic University of Rio de Janeiro, 22541-041 Rio de Janeiro, Brazil
* Correspondence: mlpacheco@fc.ul.pt

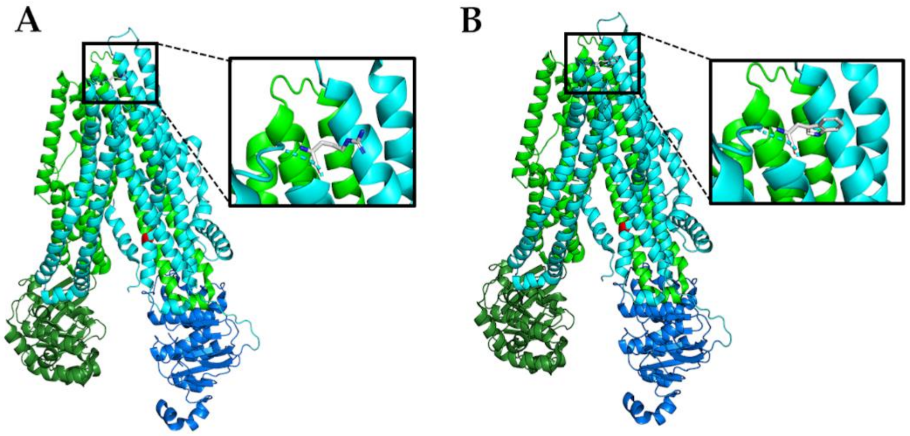

Figure S1. Ribbon diagram of the dephosphorylated, ATP-free human CFTR structure (PDB: 5UAK) demonstrating the location of the R334 residue in WT-CFTR (**A**) and mutant R334W-CFTR (**B**).

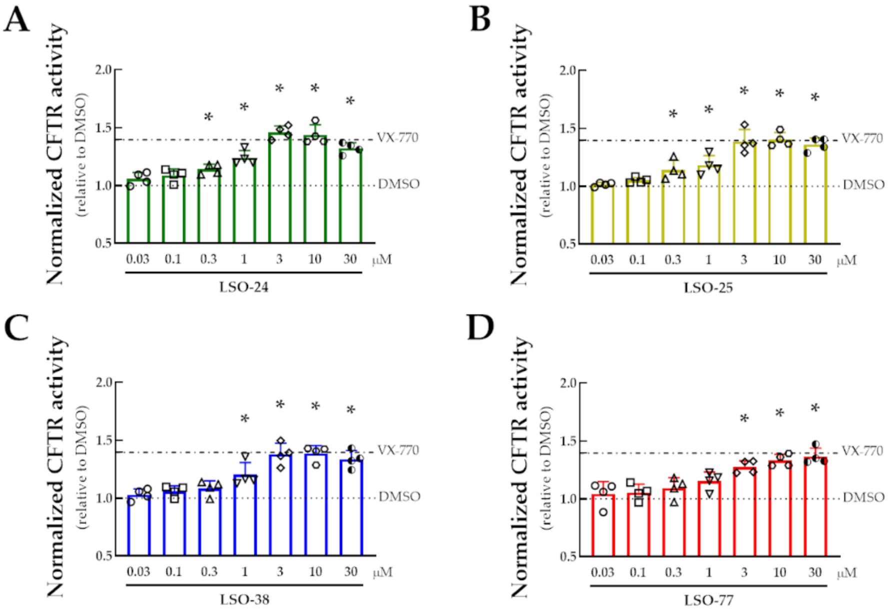

Figure S2. Dose-response relationships for the compounds (**A**) LSO-24, (**B**) LSO-25, (**C**) LSO-38 and (**D**) LSO-77 were determined by the HS-YFP assay on a plate reader in CFBE cells expressing R334W-CFTR. *P < 0.05 *vs.* DMSO.

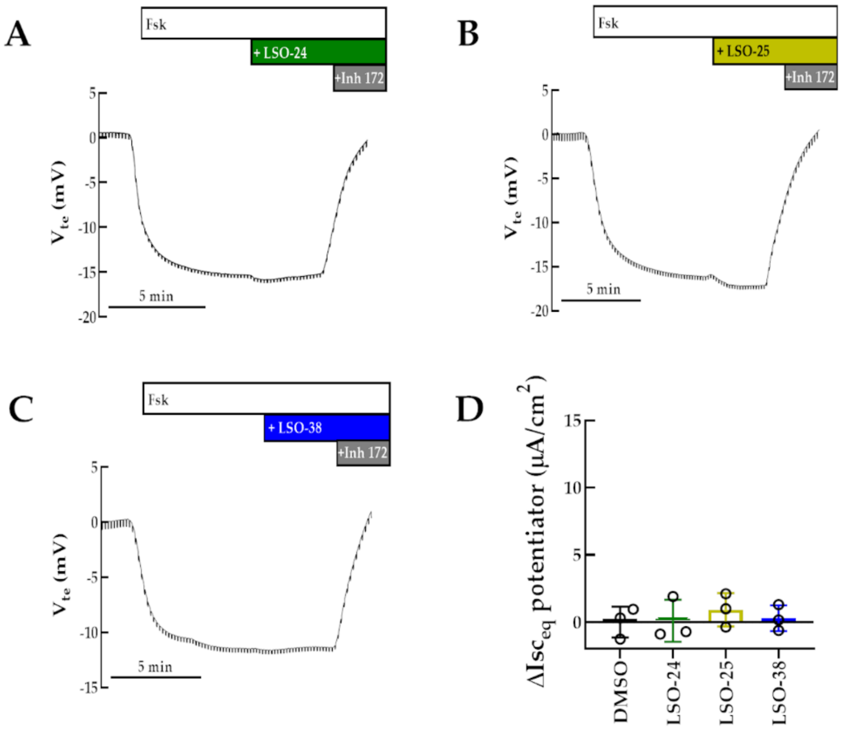

Figure S3. (**A–C**) Representative Ussing chamber (open-circuit) recording representing transepithelial voltage measurements (V_{te}) of polarized monolayers of CFBE cells expressing WT-CFTR. Cells were acutely subsequently stimulated with Fsk (0.128 µM), test compounds (DMSO, 5 µM LSO-24, 5 µM LSO-25 or 5 µM LSO-38) and CFTRInh-172 (30 µM). (**D**) Data are represented as mean (+ SD) increase in I_{eq} promoted by test potentiator.
